# Nematode-Induced Interference with Vaccination Efficacy Targets Follicular T Helper Cell Induction and Is Preserved after Termination of Infection

**DOI:** 10.1371/journal.pntd.0003170

**Published:** 2014-09-25

**Authors:** Irma Haben, Wiebke Hartmann, Minka Breloer

**Affiliations:** Bernhard Nocht Institute for Tropical Medicine, Hamburg, Germany; Uniformed Services University of the Health Sciences, United States of America

## Abstract

One-third of the human population is infected with parasitic worms. To avoid being eliminated, these parasites actively dampen the immune response of their hosts. This immune modulation also suppresses immune responses to third-party antigens such as vaccines. Here, we used *Litomosoides sigmodontis*-infected BALB/c mice to analyse nematode-induced interference with vaccination. Chronic nematode infection led to complete suppression of the humoral response to thymus-dependent vaccination. Thereby the numbers of antigen-specific B cells as well as the serum immunoglobulin (Ig) G titres were reduced. T_H_2-associated IgG1 and T_H_1-associated IgG2 responses were both suppressed. Thus, nematode infection did not bias responses towards a T_H_2 response, but interfered with Ig responses in general. We provide evidence that this suppression indirectly targeted B cells via accessory T cells as number and frequency of vaccine-induced follicular B helper T cells were reduced. Moreover, vaccination using model antigens that stimulate Ig response independently of T helper cells was functional in nematode-infected mice. Using depletion experiments, we show that CD4^+^Foxp3^+^ regulatory T cells did not mediate the suppression of Ig response during chronic nematode infection. Suppression was induced by fourth stage larvae, immature adults and mature adults, and increased with the duration of the infection. By contrast, isolated microfilariae increased IgG2a responses to vaccination. This pro-inflammatory effect of microfilariae was overruled by the simultaneous presence of adults. Strikingly, a reduced humoral response was still observed if vaccination was performed more than 16 weeks after termination of *L. sigmodontis* infection. In summary, our results suggest that vaccination may not only fail in helminth-infected individuals, but also in individuals with a history of previous helminth infections.

## Author Summary

Parasitic worms, called *helminths*, infect one-third of the world population. Despite exposure to their host's immune system many helminths establish chronic infections and survive several years within their host. They avoid elimination by dampening the immune response of their hosts. This immune suppression also affects immune responses to third-party antigens such as vaccines. Indeed, accumulating evidence suggests that helminth-infected humans display impaired responses to vaccination. Thus, anthelminthic treatment before vaccination is discussed. Here, we use helminth-infected mice to analyse kinetics and mechanism of helminth-induced interference with vaccination efficacy more precisely. We show that chronic helminth infection completely suppressed antibody responses to a model vaccine. Thereby helminths suppressed the antibody-producing B cells indirectly via suppression of accessory T helper cells. The suppression was more pronounced at later time points of infection and still observed in mice that had terminated the helminth infection for more than 16 weeks. In summary, our results suggest that vaccination may not only fail in helminth-infected individuals, but also in individuals with a history of previous helminth infections. Thus, our report highlights the importance to develop vaccination strategies that are functional despite concurrent helminth infection rather than deworming humans before vaccination.

## Introduction

More than 1 billion people are infected with helminths worldwide, predominantly in the tropics and subtropics [1]. To avoid their elimination and to limit pathology, helminths have developed sophisticated strategies to dampen the immune response of their hosts [2], [3]. This helminth-induced immune suppression also affects the immune response to third-party antigens and thus may interfere with efficient response to co-infecting pathogens and to vaccination [4]. Reduced cellular and humoral responses have been observed in helminth-infected humans after cholera [5], Bacillus Calmette-Guerin (BCG) [6], [7], tetanus toxoid [8], [9], [10], [11], [12] and anti-*Plasmodium falciparum* vaccination [13] (reviewed in [14], [15], [16]). Drug-induced termination of helminth infection improved responses to BCG [6], [17] and cholera [5], [18] vaccination. These collective studies emphasize that in addition to the pathology caused by helminth infection itself, helminth-induced interference with vaccination efficacy represent a global health problem.

As the nature of human studies is predominantly descriptive, murine models of helminth infection have been established to further investigate these issues. Responses to HIV or BCG vaccination were compromised in mice infected with the pathogenic trematode *Schistosoma mansoni* [19], [20]; similarly infection with the gastrointestinal nematode *Heligmosomoides polygyrus* interfered with humoral and cellular responses to malaria vaccinations [21], [22]. The nematode *Litomosoides sigmodontis* is used to model human filarial infections [23], [24]. *L. sigmodontis* third stage larvae (L3) are transmitted during the blood meal by the arthropod intermediate host, the mite *Ornithonyssus bacoti*. The natural definitive host is the cotton rat *Sigmodon hispidus*; however, some laboratory mouse strains such as BALB/c mice are fully susceptible to infection. L3 migrate during the first three days via the lymphatic system to the thoracic cavity. They moult to fourth stage larvae (L4) within 10 days and to immature adults within 30 days. Mature adults mate around day 55 post-infection (p.i.) and females release first stage larvae known as microfilariae (MF) into the peripheral circulation by day 60 p.i. Although BALB/c mice eventually control parasite burden by innate and adaptive immunity, they remain infected for several months until the parasites are fully eradicated. Thus, *L. sigmodontis*-infected BALB/c mice provide a suitable model to analyse the impact of chronic nematode infections on co-infections [25], [26], autoimmune diseases [27], [28] and allergy [29].

We used *L. sigmodontis* infection to study the effect of concurrent nematode infection on vaccination efficacy. An experimental vaccination against the liver stage of *Plasmodium berghei* using a single injection of a *P. berghei* circumsporozoite (CSP) fusion protein induced lower numbers of CSP-specific CD8^+^ cytotoxic T cells, if vaccinated mice were infected with *L. sigmodontis* [30]. Using semi-resistant C57BL/6 mice we have shown that acute *L. sigmodontis* infection suppressed humoral responses to thymus-dependent (TD) model antigen vaccinations performed at day 14 of infection [31]. Since C57BL/6 mice terminate *L. sigmodontis* infection around day 60 before patency is established [32] the impact of chronic nematode infection can not be modelled using this mouse strain.

In the current study we use the fully susceptible BALB/c mice to analyse the impact of different *L. sigmodontis* life stages on vaccination efficacy. We report an almost complete suppression of IgG response to TD vaccination in chronically infected mice. Suppression was induced by L4 and by parasitic adults that outcompeted the pro-inflammatory effect stimulated by MF. Suppression was observed even when vaccination was performed several months after termination of *L. sigmodontis* infection. We provide evidence that suppression of TD response during chronic nematode infection was established by interference with follicular B helper T cell (T_FH_) induction, independent of Foxp3^+^ T_reg_.

## Methods

### Ethics statement

Animal experimentation was conducted at the animal facility of the Bernhard Nocht Institute for Tropical Medicine (BNITM) in agreement with the German animal protection law under the supervision of a veterinarian. The experimental protocols have been reviewed and approved by the responsible Federal Health Authorities of the State of Hamburg, Germany, the “Behörde für Gesundheit und Verbraucherschutz” permission number 98/11. BALB/c mice, cotton rats (*S. hispidus*) and BALB/c “Depletion of Regulatory T cell” (DEREG) mice were bred in the animal facilities of the BNITM and kept in individually ventilated cages under specific pathogen-free conditions. Mice were sacrificed by deep CO_2_ narcosis.

### *L. sigmodontis* cycle and experimental infections

The life cycle of *L. sigmodontis* was maintained as described [31]. Six- to eight-week-old female mice were naturally infected by exposure to *L. sigmodontis*-infected mites *(O. bacoti)* that transmit infectious L3 during the blood meal. L4, adult worms and granulomata were harvested by flushing the thoracic cavity of infected mice with 8–10 mL of PBS. Parasites were counted subsequently. To detect MF in the circulation, blood of infected mice was collected in EDTA tubes; subsequently 20 µL of blood was added to 100 µL of ddH_2_O, and centrifuged at 10,000× g for 5 min. The pellet was resolved in 20 µL of Gentian violet and all MF were counted.

### Isolation of MF and treatment of mice with MF

*L. sigmodontis* MF were purified from blood of infected cotton rats by density gradient centrifugation on Percoll. EDTA blood of cotton rats was collected and diluted 1∶2 with PBS. Iso-osmotic Percoll (Sigma-Aldrich, Munich, Germany) was prepared by mixing 9 parts of Percoll (density, 1.130 g/mL) with 1 part of 2.5 M Sucrose (Sigma-Aldrich, Munich, Germany). The following dilutions of 90% Percoll in 0.25 M Sucrose were made: 25% and 30% and layered with the diluted blood on top. After centrifugation at 400× g for 30 min at room temperature (RT) without brakes, MF are located between the 25% and 30% layer. MF were harvested, washed twice with PBS by 30 min centrifugation at 400× g and counted. Mice received 10,000 viable MF i.v.

### Model Ag vaccination

Non-infected and *L. sigmodontis*-infected mice were vaccinated at indicated time points post infection by i.p. injection of either 100 µg alum-precipitated dinitrophenol-keyhole limpet hemocyanin (DNP-KLH, Sigma-Aldrich, Munich, Germany), 100 µg 4-hydroxy-3-iodo-5-nitrophenylacetyl (NIP) conjugated to Ficoll (NIP-Ficoll) (Biosearch Technologies, Navato, USA), or by s.c. injection of 30 µg alum-precipitated DNP-KLH into the hind footpad. For analysis of serum antibodies, blood was collected from mice by submandibular bleeding of the facial vein 7, 14 and 21 days after vaccination and allowed to coagulate for 1 h at RT. Serum was collected after centrifugation at 10,000× g for 10 min at RT and stored at −20°C for further analysis. For analysis of spatial separated cellular responses, mice were sacrificed at the indicated time point, and spleen and popliteal lymph nodes (popLN) were dissected. A total of 2.5×10^5^ splenocytes or popLN cells were cultured in 3–5 replicates in 96-well round-bottom plates in RPMI 1640 medium supplemented with 10% FCS, 20 mM HEPES, 2 mM L-glutamine and gentamicin (50 µg/mL) at 37°C and 5% CO_2_. The supernatant was harvested after 21 days of culture and DNP- and *L. sigmodontis*-specific IgG1, IgG2a and IgG2b were quantified by ELISA.

### Quantification of humoral response by ELISA

DNP-KLH was chosen as TD model antigen as no cross-reaction between DNP-specific Ig and *L. sigmodontis* antigen was detected and comparison of DNP_7_-BSA and DNP_38_-BSA allows detection of high affinity only as well as high and low affinity Ig. For the detection of DNP-, NIP- and *L. sigmodontis*-specific Ig, ELISA plates were coated overnight with 1 µg/mL DNP_7_-BSA, (Sigma-Aldrich, Munich, Germany), 1 µg/mL NIP_7_-BSA (Biosearch Technologies, Navato, USA), or 4 µg/mL *L. sigmodontis* extract in carbonate buffer pH 9.6. Low affinity DNP-specific IgG was detected by coating ELISA plates with 1 µg/mL DNP_38_-BSA (Biotrend Chemikalien, Cologne, Germany). Plates were washed, blocked by incubation with PBS 1% BSA for 2 h and incubated for 2 h with serum or cell culture supernatant. Plates were washed and incubated for 1 h with horseradish peroxidase-labelled anti-mouse IgM, IgG1, IgG2a, IgG2b (Life Technologies, Carlsbad, USA), or IgG3 (Southern Biotechnology Associates, Birmingham, USA). Plates were washed and developed by incubation with 100 µL tetramethylbenzidine (0.1 mg/mL), 0.003% H_2_O_2_ in 100 mM NaH_2_PO_4_ pH 5.5 for 2.5 min. Reaction was stopped by addition of 25 µL 2 M H_2_SO_4_, and OD_450_ was measured. For the more abundantly produced isotypes IgG1 and IgM, titres were calculated by defining the highest serum dilution in a serial dilution (1∶1000 to 1∶128,000) resulting in an OD_450_ above the doubled background. For the less abundant isotypes IgG2a, IgG2b, and IgG3, arbitrary units were calculated by subtraction of OD_450_ of the background from OD_450_ of one fixed serum concentration (1∶100 for IgG2a, 1∶1000 for IgG2b, and 1∶100 for IgG3). Background was generally below OD_450 = _0.1. Calculation of titres by serial dilution for random control samples revealed similar differences in DNP-specific IgG2a and IgG2b production as the arbitrary units and thus did not yield additional information (data not shown).

### Flow cytometry

For analysis of B and T_FH_ cells, mice were sacrificed at the indicated time points and popLN were dissected. Cells (1×10^6^) were stained with Live/Dead Fixable Blue Dead Cell Stain Kit (Life Technologies, Carlsbad, USA) according to the manufacturer's instructions. For surface staining, cells were stained with anti-CXCR5-Biotin (clone: 2G8) for 30 min at 37°C or with anti-IgG-phycoerythrin-Cy7 (PE-Cy7) (clone: Poly4053) and Biotin-labelled peanut agglutinin (PNA) (Galab Technologies, Geesthacht, Germany) for 30 min at 4°C. After washing the cells were stained with anti-CD3e-allophycocyanin (APC) (145-2C11), anti-CD4-APC/Brilliant Violet 510 (BV510)/PE (clone: RM4-5), anti-CD44-BV421 (clone: IM7), anti-PD1-fluorescein isothiocyanate (FITC) (clone: 29F.1A12), anti-ICOS-PE (clone: 7E.17G9), anti-CD19-PE (clone: 6D5), DNP-BSA-AF647, anti-IgM-FITC (clone: DS1), Strepavidin-APC and Strepavidin-BV421 for 30 min at 4°C. Foxp3 expression was determined using PE-anti-mouse Foxp3 Staining Set (clone: FJK-16S, Affymetrix eBioscience, Frankfurt, Germany) according to the manufacturer's instructions. Foxp3^+^ T_reg_ depletion was controlled by analysis of eGFP, Foxp3 and CD4 expression. Samples were analysed on a LSR II Flow Cytometer (Becton Dickinson, Mountain View, USA) using FlowJo software (TreeStar, Ashland, USA). DNP-BSA was fluorescence labelled using Alexa Fluor 647 Protein Labeling Kit (Life Technologies, Carlsbad, USA) according to the manufacturer's instructions. Unless otherwise stated all staining antibodies were purchased from BioLegend (Fell, Germany), BD Biosciences (Heidelberg, Germany) or Affymetrix eBioscience (Frankfurt, Germany).

### *In vivo* depletion of T_reg_

Heterozygous BALB/c DEREG mice and non-transgenic littermate control BALB/c mice received 0.5 µg diphtheria toxin (DT) (Merck, Darmstadt, Germany) dissolved in PBS i.p. on three consecutive days, starting either two days prior to *L. sigmodontis* infection or one day before vaccination. Successful depletion of Foxp3^+^ Treg was routinely controlled by staining for CD4^+^Foxp3^+^ cells in the peripheral blood after the third DT treatment.

### Statistical analysis

Statistical analysis was performed by ANOVA with Bonferroni post-test or student's t-test using Prism software (GraphPad Software, San Diego, USA). Results are presented as mean ± SEM; p≤0.05 was considered statistically significant.

## Results

### Chronic nematode infection suppresses humoral response to TD model antigen vaccination

**Figure 1:**
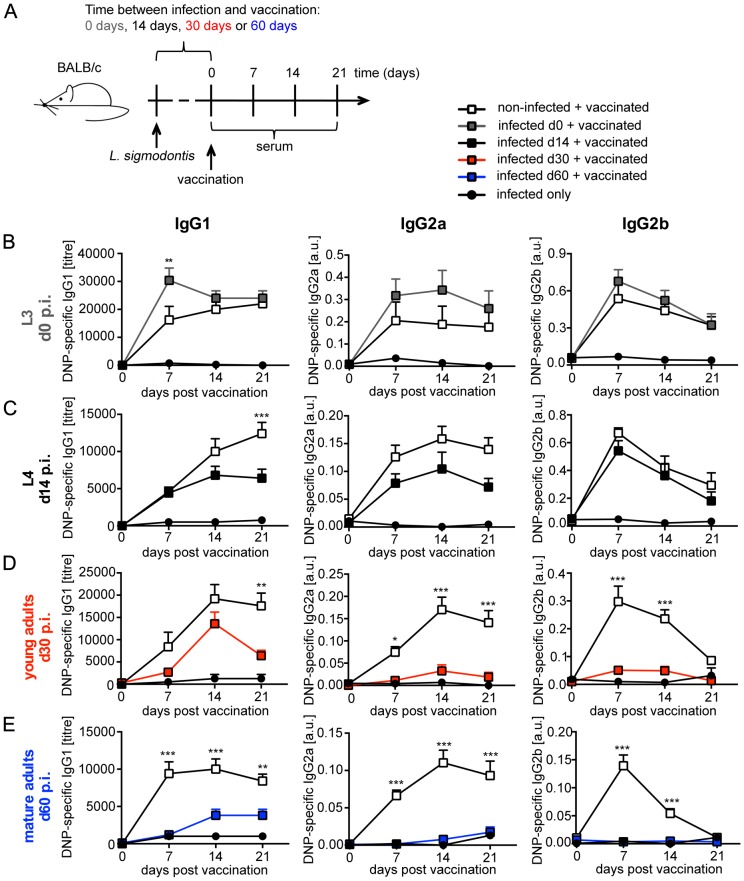
Infection with *L. sigmodontis* suppresses humoral response to TD vaccination. **(A)** Diagram of the experimental setup. Six- to eight-week-old BALB/c mice were naturally infected with *L. sigmodontis* (closed squares) or left non-infected (open squares; n = 10) and were vaccinated with 100 µg DNP-KLH/Alum i.p. at **(B)** day 0 (grey, n = 8–10), **(C)**) day 14 (black, n = 10), **(D)** day 30 (red, n = 10) or **(E)** day 60 (blue, n = 10) p.i. An additional control group was infected but not vaccinated (closed circles, n = 2–4). DNP-specific IgG1, IgG2a and IgG2b was detected in sera. Results are expressed as mean ± SEM of pooled data derived from two independent experiments. Asterisks indicate significant differences of the mean of DNP-specific Ig in non-infected and infected mice after vaccination with DNP-KLH (Two-way ANOVA).

We used BALB/c mice that are fully susceptible for *L. sigmodontis* infection to analyse the impact of different parasitic life stages on vaccination efficacy. By choosing different durations between exposure to infected mites and subsequent vaccination we defined the life stage of *L. sigmodontis* present at the moment of vaccination (Figure 1A). We analysed the impact of L3 migrating via the lymphatic vessels to the thoracic cavity by vaccinating mice at the day of infection. To analyse the impact of L4 and young immature adults on vaccination efficacy, we vaccinated 14 and 30 days after infection. Vaccination at day 60 p.i. resulted in the presence of mature adults that had mated and released MF into the peripheral circulation.

We employed alum-precipitated DNP-KLH as a model vaccine for TD humoral response. High affinity DNP-specific IgG responses were analysed in nematode-infected and in age-matched non-infected control mice on three consecutive weeks following vaccination. Simultaneous nematode infection increased early responses to DNP-KLH vaccination (Figure 1B). In contrast, presence of *L. sigmodontis* L4 during vaccination reduced DNP-specific IgG1, whereas DNP-specific IgG2a was reduced by trend and IgG2b was unchanged (Figure 1C). Vaccination at later time points of infection, i.e. day 30 or day 60 p.i., resulted in statistically significant suppression of IgG1, IgG2a, and IgG2b responses to DNP-KLH, in comparison to age-matched non-infected mice (Figure 1D,E). DNP-specific IgG2 response was almost absent in day 60 infected mice.

The capture agent used in these experiments, i.e. BSA coupled to 7 DNP molecules, was suited to detect specifically high affinity IgG. Re-analysis of these sera with BSA coupled to 38 DNP molecules, a setting that will capture IgG displaying a lower affinity to DNP in addition to high affinity DNP-specific IgG, revealed similar results (Figure S1 in Text S1). Thus, the quantity but not the quality of IgG response to vaccination was reduced by established nematode infection.

**Figure 2:**
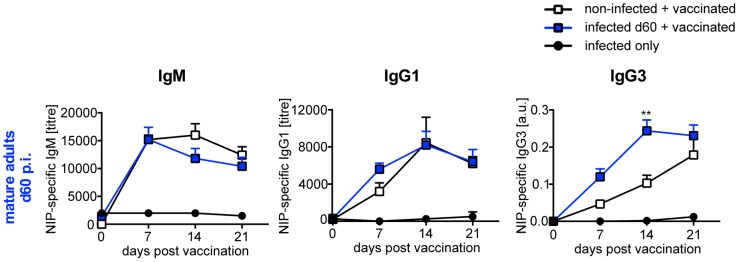
Infection with *L. sigmodontis* does not suppress humoral response to TI vaccination. Six- to eight-week-old BALB/c mice were naturally infected with *L. sigmodontis* (blue squares, n = 10) or left non-infected (white squares, n = 10) and vaccinated with 100 µg NIP-Ficoll i.p. at day 60 p.i. An additional control group was infected but not vaccinated (closed circles, n = 2). NIP-specific IgM, IgG1 and IgG3 was quantified in sera. Results are expressed as mean ± SEM of pooled data derived from two independent experiments. Asterisks indicate significant differences of the mean of NIP-specific Ig in non-infected and infected mice after vaccination with NIP-Ficoll (Two-way ANOVA).

In line with our previous results [31], [33], nematode infection did not interfere with the humoral response to the polyvalent TI model antigen NIP-Ficoll that activates B cells in the absence of T helper cells by strong crosslinking of the B cell receptor. Due to absent T cell co-stimulation NIP-Ficoll induces predominantly IgM responses and limited IgG1 and IgG3 responses [34]. Consequently we did not detect NIP-specific IgG2a or IgG2b in NIP-Ficoll vaccinated mice (data not shown) but NIP-specific IgM, IgG1 and IgG3 were produced (Figure 2). Thereby, NIP-specific humoral responses were similar, or in the case of IgG3, even increased in NIP-Ficoll-vaccinated non-infected in comparison to day 60 *L. sigmodontis*-infected mice.

Taken together, these results show that the presence of L4 and adult *L. sigmodontis*, but not of recently transmitted L3, suppressed humoral response to vaccination specifically in T cell-dependent settings. Intensity of suppression was positively correlated to duration of nematode infection. Chronic nematode infection suppressed both, T_H_2-associated IgG1 and T_H_1-associated IgG2 responses to vaccination, thus, inflicting generalized suppression and not polarization towards a type 2 immune response.

Despite the apparent suppression of TD humoral response in infected mice, *L. sigmodontis*-specific IgG1, IgG2a and IgG2b responses were detectable during infection (Figure S3 in Text S1). Additional DNP-KLH vaccination did not modulate the *L. sigmodontis*-specific Ig response, as expected (Figure S3 in Text S1: black circles and grey squares).

### *L. sigmodontis* adults silence the pro-inflammatory effect of isolated MF

**Figure 3:**
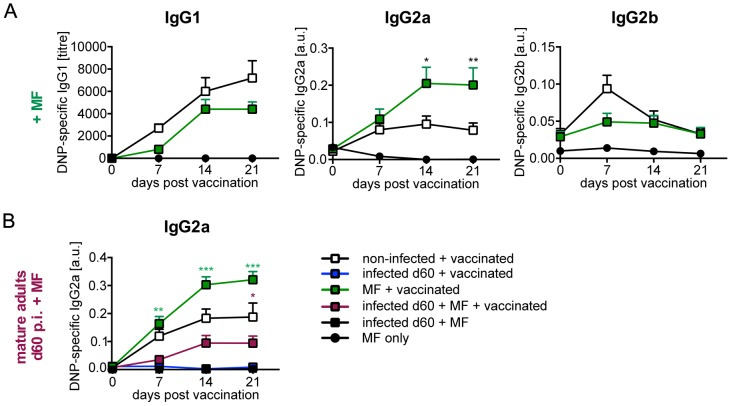
Opposing impact of *L. sigmodontis* adults and MF on humoral response to TD vaccination. **(A)** Six- to eight-week-old BALB/c mice received 10,000 MF i.v. (green squares, n = 10) or were left untreated (white squares, n = 10). Both groups were vaccinated with 100 µg DNP-KLH/Alum i.p. immediately. Control mice (black circles, n = 2) received 10,000 MF i.v. but were not immunized. DNP-specific IgG1, IgG2a and IgG2b was quantified in sera. Results are expressed as mean ± SEM of pooled data derived from two independent experiments. Asterisks indicate significant differences of the mean of DNP-specific Ig in non-treated and MF-treated mice (Two-way ANOVA). **(B)** Six- to eight-week-old BALB/c mice were naturally infected with *L. sigmodontis* (closed squares) or left non-infected (white squares, n = 8). Day 60 *L. sigmodontis*-infected mice received 10,000 MF i.v. additionally (purple squares, n = 10) and all groups were vaccinated with 100 µg DNP-KLH/Alum i.p. immediately. Control mice were also vaccinated with 100 µg DNP-KLH/Alum i.p. and either infected for 60 days (blue squares, n = 2) or received purified MF only (green squares, n = 4). Non-vaccinated control mice were infected for 60 days and received MF i.v. (black squares, n = 2) or just received MF i.v. (black circles, n = 2). DNP-specific IgG2a was quantified in sera. Results are expressed as mean ± SEM of pooled data derived from two experiments. Black asterisks indicate significant differences of the mean of DNP-specific Ig in non-infected and infected BALB/c mice. Purple asterisks indicate significant differences of the mean of DNP-specific Ig in non-infected and infected MF-treated mice. Green asterisks indicate significant differences of the mean of DNP-specific Ig in MF-treated non-infected and MF-treated day 60 *L. sigmodontis*-infected mice (student's t-test).

Between 40 and 60% of infected BALB/c mice developed detectable microfilaraemia by day 60 p.i., leading to simultaneous presence of two different life stages in mice vaccinated at this late time point. Stratification of DNP-specific IgG response of day 60 infected mice that were positive (n = 7) or negative (n = 12) for MF in the peripheral circulation revealed no differences in suppression (Figure S2 in Text S1). To differentiate between the impact of adults and MF on response to DNP-KLH vaccination, we injected 10,000 purified MF at the day of vaccination to model the recent release of MF by females. Interestingly, presence of isolated MF increased the IgG2a response to vaccination while the IgG1 and IgG2b responses remained unchanged (Figure 3A). Thus, MF displayed a pro-inflammatory effect, increasing T_H_1-associated Ig responses to third-party antigens. This finding suggests that the anti-inflammatory effect observed in day 60 *L. sigmodontis*-infected microfilaraemic mice was induced by adults and outcompeted the pronounced pro-inflammatory effect of isolated MF.

However, injection of 10,000 MF in a bolus may trigger stronger pro-inflammatory signals than the putative effects mediated by MF released gradually by female adults *in vivo*. To investigate if adults would also suppress the possibly stronger pro-inflammatory signals delivered by isolated MF we injected purified MF into day 60 *L. sigmodontis*-infected mice (Figure 3B). While injection of MF into non-infected mice increased IgG2a responses compared to naïve mice as observed before, MF-treated *L. sigmodontis*-infected mice displayed reduced IgG2a responses in comparison to MF-treated non-infected mice. IgG response in MF-treated *L. sigmodontis*-infected mice was also significantly lower than IgG response in non-infected mice. Taken together, these results show that adults suppress the IgG response to vaccination in the presence of circulating MF, despite pro-inflammatory stimuli transduced by MF.

### Foxp3^+^ T_reg_ are dispensable for suppression of humoral response to TD vaccination during chronic *L. sigmodontis* infection

**Figure 4:**
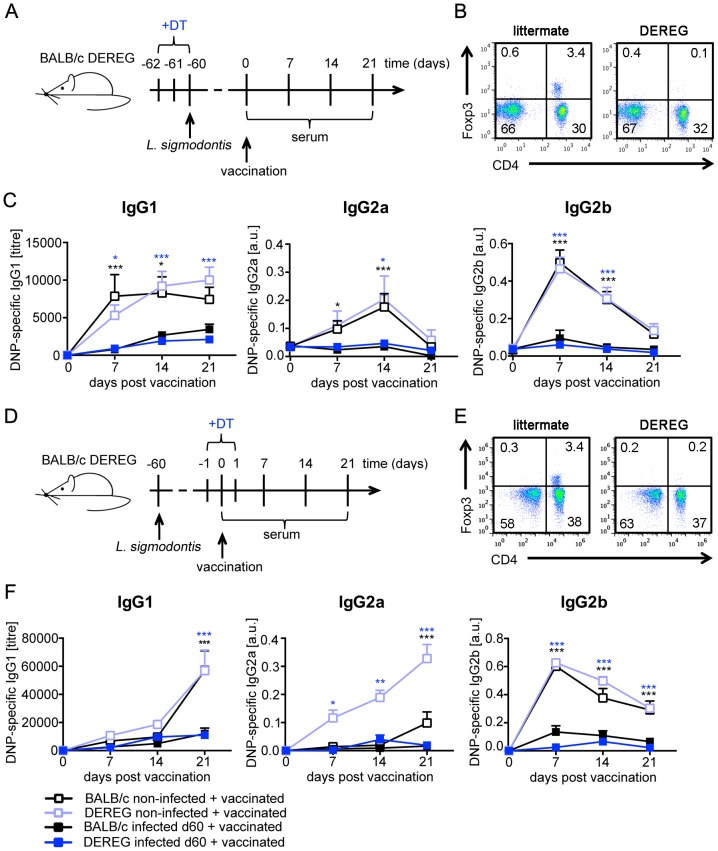
Foxp3^+^ Treg do not mediate suppression of humoral response in *L. sigmodontis*-infected mice. **(A)** Diagram of the experimental setup. Six- to eight-week-old BALB/c DEREG mice and littermates were naturally infected with *L. sigmodontis* or left non-infected. To deplete T_reg_, all mice received diphtheria toxin (DT, 0.5 µg i.p.) on three consecutive days starting two days before infection. At day 60 p.i. mice were vaccinated with 100 µg DNP-KLH/Alum i.p. **(BE)** Peripheral blood lymphocytes were stained for CD4 and Foxp3 to control depletion of T_reg_ at the day of the third DT injection. **(C)** Quantification of DNP-specific IgG1, IgG2a and IgG2b in sera of DNP-KLH-vaccinated non-infected BALB/c (white squares, n = 7) and DEREG (light blue squares, n = 10) and *L. sigmodontis*-infected BALB/c (black squares, n = 9) and DEREG (dark blue squares, n = 9) mice. **(D)** Diagram of the experimental setup. Six- to eight-week-old BALB/c DEREG mice and littermates were naturally infected with *L. sigmodontis* or left non-infected. To deplete T_reg_, all mice received DT (0.5 µg i.p.) on three consecutive days starting one day before vaccination. At day 60 p.i. mice were vaccinated with 100 µg DNP-KLH/Alum i.p. **(F)** Quantification of DNP-specific IgG1, IgG2a and IgG2b in sera of DNP-KLH-vaccinated non-infected BALB/c (white squares, n = 7) and DEREG (light blue squares, n = 9), and *L. sigmodontis*-infected BALB/c (black squares, n = 9) and DEREG (dark blue squares, n = 6) mice. Results are expressed as mean ± SEM of pooled data derived from two independent experiments. Black asterisks indicate significant differences of the mean of DNP-specific Ig in non-infected and infected BALB/c mice. Blue asterisks indicate significant differences of the mean of DNP-specific Ig in non-infected and infected DEREG mice (Two-way ANOVA).

Foxp3^+^ T_reg_ are central regulators of adaptive immune responses and have been shown to mediate helminth-induced immune suppression [35]. Although our previous study did not indicate a function for Foxp3^+^ T_reg_ in the suppression of CD4^+^ T cell proliferation during acute *L. sigmodontis* infection in C57BL/6 mice [31], accumulating evidence suggests that the dominance of T_reg_-mediated regulation differs in different mouse strains [36], [37], [38], [39]. Therefore we evaluated the contribution of Foxp3^+^ T_reg_ to the suppression of vaccination efficacy in day 60 *L. sigmodontis*-infected BALB/c mice. To this end, we employed BALB/c DEREG mice that express a fusion protein consisting out of the human diphtheria toxin (DT) receptor and enhanced green fluorescent protein (eGFP) under the control of the Foxp3 promoter [40]. Injection of DT results into transient depletion of Foxp3^+^CD4^+^ T cells in DEREG mice while T_reg_ frequencies in non-transgenic littermates remain unchanged [41]. As Foxp3^+^ T_reg_ depletion is not permanent in DEREG mice, we investigated the effect of transient Foxp3^+^ T_reg_ depletion either during initial infection (Figure 4A) or during vaccination of chronically infected mice (Figure 4D).

Absence of Foxp3^+^ T_reg_ during the first days of *L. sigmodontis* infection did not abrogate suppression of IgG response to vaccination in nematode-infected BALB/c mice (Figure 4C). DNP-specific IgG1, IgG2a and IgG2b responses were equally reduced in nematode-infected mice containing Foxp3^+^ T_reg_ (black squares) or not containing Foxp3^+^ T_reg_ (blue squares). Successful depletion of Foxp3^+^ T_reg_ was verified by flow cytometry at the day of *L. sigmodontis* infection (Figure 4B).

Transient T_reg_ depletion at the moment of vaccination (Figure 4D) that was confirmed by flow cytometry one day after vaccination (Figure 4E) increased the DNP-specific IgG2a response in non-infected mice (Figure 4F). Similar increases in pro-inflammatory IgG2a responses upon T_reg_ depletion were observed in a recent study using DEREG mice in a model of atopic dermatitis [42]. Increased IgG2a responses reflected most likely inefficient regulation due to the absence of T_reg_ and, thus function as internal control for depletion efficacy. Nevertheless, increased DNP-specific IgG2a in T_reg_-depleted mice was still suppressed upon *L. sigmodontis* infection. T_reg_ depletion during vaccination did not modulate the more abundant IgG1 or IgG2b responses and did not abrogate nematode-induced suppression of IgG response to vaccination. Taken together, these results rule out a contribution of Foxp3^+^ T_reg_ to the suppression of IgG response to TD vaccination in nematode-infected BALB/c mice.

### Suppression of humoral response to TD vaccination during chronic *L. sigmodontis* infection is not due to competition of nematode-specific and vaccination-induced lymphocytes

**Figure 5:**
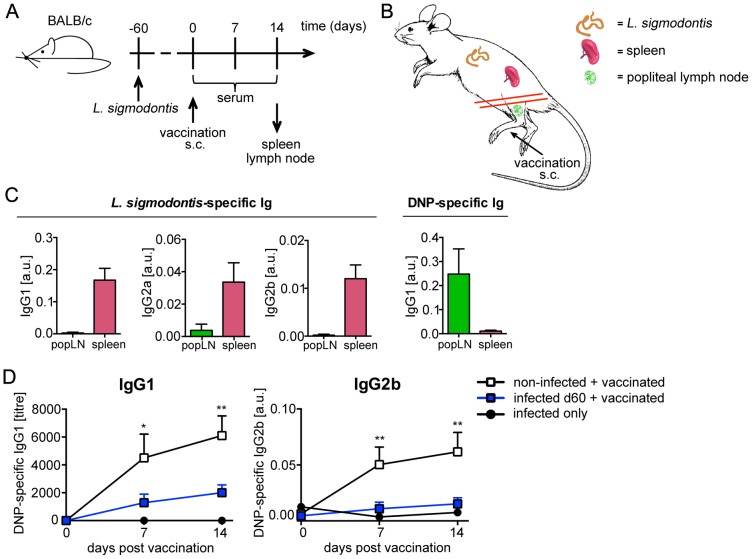
Spatial separation of nematode- and vaccine-specific responses. **(A)** Diagram of the experimental setup. Six- to eight-week-old BALB/c mice were naturally infected with *L. sigmodontis* or left non-infected and vaccinated with 30 µg DNP-KLH/Alum s.c. at day 60 p.i. **(B)** Diagram of spatial separation of immune responses. **(C)** Splenocytes and draining popLN cells were cultured for 21 days at 37°C. Supernatants were analysed for DNP-specific and *L. sigmodontis*-specific IgG1, IgG2a and IgG2b. **(D)** Quantification of DNP-specific IgG1 and IgG2b in sera of *L. sigmodontis*-infected (blue squares, n = 14) and non-infected (white squares, n = 10) vaccinated mice. Control mice were infected but not vaccinated (black circles, n = 2). Results are expressed as mean ± SEM of pooled data derived from two independent experiments. Asterisks indicate significant differences of the mean of DNP-specific Ig in non-infected and infected mice (Two-way ANOVA).

Natural infection with *L. sigmodontis* that predominantly dwell in the thoracic cavity induced a systemic immune response. Nematode-specific T and B cell responses are detectable in the draining lymph nodes and in the spleen (data not shown). In the experiments performed so far, mice were vaccinated i.p., thereby inducing systemic responses to DNP-KLH that are initiated mostly in the spleen. To rule out that suppression of IgG response to vaccination was caused by a simple competition of nematode- and vaccine-specific B and T cells in the same lymphatic organ, we separated the sites of *L. sigmodontis*-specific and DNP-KLH-specific immune responses (Figure 5).

To this end we vaccinated day 60 *L. sigmodontis*-infected and age-matched non-infected mice with DNP-KLH subcutaneously (s.c.) into the hind footpad. This regimen is suited to induce DNP-KLH-specific T and B cell responses predominantly in the draining popliteal lymph node (Figure 5AB). Fully differentiated plasma cells will predominantly migrate into the bone marrow and secrete Ig into the peripheral circulation. The induction of humoral responses in lymph nodes or in the spleen can be visualized by presence of B cells that secrete Ig spontaneously at low concentrations in cell cultures of these lymphatic organs. Cultured splenocytes derived from vaccinated and *L. sigmodontis*-infected mice secreted nematode-specific IgG1, IgG2a and IgG2b but did not secrete any DNP-specific IgG (Figure 5C). Cultured popliteal lymph node cells derived from the same mice produced DNP-specific but not nematode-specific IgG1, thus visualizing the spatial separation of B cell responses to parasite and vaccine. DNP-specific IgG2a and IgG2b concentrations in the culture supernatant were below the detection limit. Systemic serum titres of DNP-specific IgG1 and IgG2b was still suppressed in day 60 *L. sigmodontis*-infected mice after s.c. vaccination (Figure 5D), demonstrating that nematode-induced suppression acted on B cell responses that were primed in a local lymph node as well. As nematode-specific lymphocytes were not present in the local lymph nodes that drained the site of DNP-KLH vaccination (Figure 5C), the observed suppression of systemic DNP-KLH-specific IgG response was not mediated by competition within the same site. Mice that were *L. sigmodontis*-infected but not vaccinated produced no DNP-specific IgG at all (Figure 1B–E and Figure 5D: black circles) and mice that were DNP-KLH vaccinated but not *L. sigmodontis*-infected did not produce any *L. sigmodontis*-specific IgG at all (Figure S3 in Text S1: white squares).

### *L. sigmodontis* infection reduces the number of DNP-KLH vaccination-induced B and T_FH_ cells

**Figure 6:**
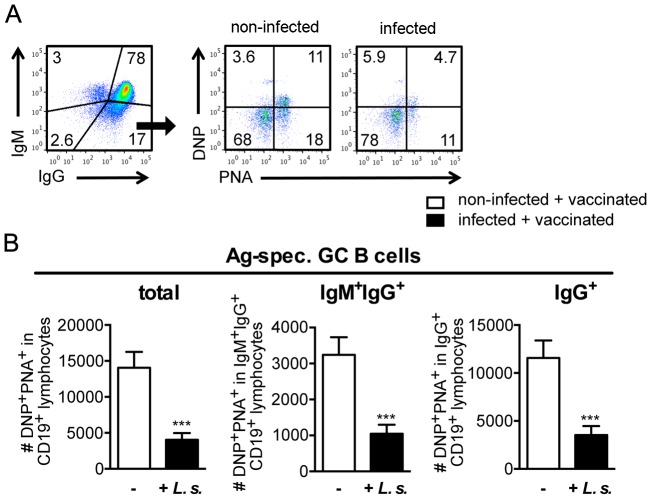
Reduced numbers of vaccine-specific B cells in *L. sigmodontis*-infected mice. Six- to eight-week-old BALB/c mice were naturally infected with *L. sigmodontis* or left non-infected and vaccinated with 30 µg DNP-KLH/Alum s.c. at day 60 p.i. PopLN cells from DNP-KLH-vaccinated non-infected (white bars) and *L. sigmodontis*-infected (black bars) mice were stained for CD19, PNA-binding, DNP-binding, IgM and IgG at day 14 post vaccination. Mice had been infected for 60 days at the time point of vaccination. **(A)** Gating strategy of CD19^+^ B lymphocytes. **(B)**Total cell numbers of DNP^+^PNA^+^ lymph node cells within the CD19^+^ (total), within the IgG^+^IgM^+^CD19^+^ and within the IgG^+^CD19^+^ gate (n = 10–14). Results are expressed as mean ± SEM of pooled data derived from two independent experiments. Asterisks indicate significant differences between non-infected and infected mice (student's t-test).

To quantify vaccine-induced B cells, we directly stained DNP-specific CD19^+^ B cells in the lymph nodes of vaccinated mice. DNP-KLH vaccination into the hind footpad induced DNP-binding CD19^+^ B cells in the draining lymph nodes (Figure 6) while no DNP-binding B cells were detectable in the non-draining contralateral lymph nodes (data not shown). DNP-specific B cells also bound peanut agglutinin (PNA) (Figure 6A), indicating localization in the germinal centre [43]. Chronic nematode infection reduced the numbers of DNP-specific PNA-binding B cells (Figure 6AB). Reduced DNP-specific B cell numbers were observed in the IgM/IgG double positive and in the terminally switched, IgG single positive B cell population. Thus, the reduced quantity of DNP-specific Ig detected in the serum of nematode-infected vaccinated mice was reflected by reduced numbers of DNP-specific B cells in the draining lymph nodes.

**Figure 7:**
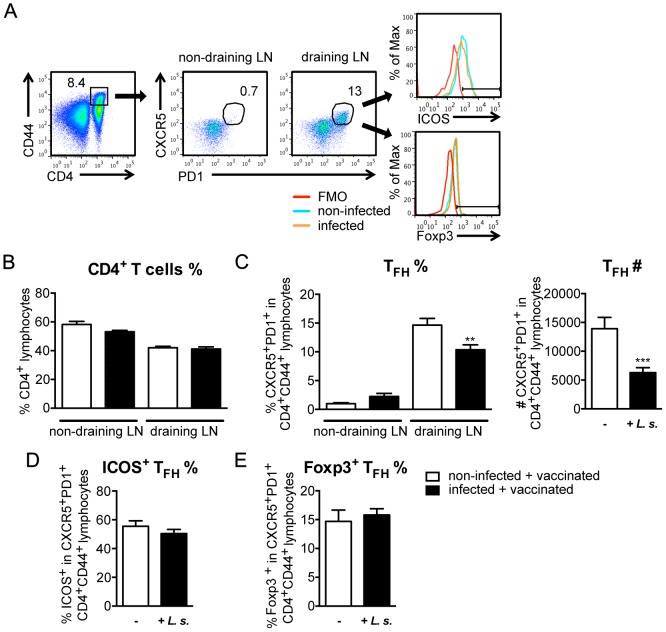
Reduced numbers and frequency of vaccine-induced T_FH_ in *L. sigmodontis*-infected mice. Six- to eight-week-old BALB/c mice were naturally infected with *L. sigmodontis* or left non-infected and vaccinated with 30 µg DNP-KLH/Alum s.c. at day 60 p.i. PopLN cells derived from DNP-KLH-vaccinated non-infected (white bars) and *L. sigmodontis*-infected (black bars) mice were stained for CD4, CD44, PD1, CXCR5, ICOS and Foxp3 at day 14 post vaccination. **(A)** Gating strategy of T_FH_. **(B)** Frequency of CD4^+^ T cells (n = 20; pooled data derived from four independent experiments). **(C)** Frequencies and total cell numbers of PD1^+^CXCR5^+^ T_FH_ cells within the CD4^+^CD44^+^ activated T helper cells (n = 14; pooled data derived from three independent experiments). **(D)** ICOS expression of PD1^+^CXCR5^+^ T_FH_ cells within the CD4^+^CD44^+^ activated T helper cells (n = 10; pooled data derived from two independent experiments). **(E)** Foxp3 expression of PD1^+^CXCR5^+^ T_FH_ cells within the CD4^+^CD44^+^ activated T helper cells (n = 5; data from one experiment representative for two independent repeats). Asterisks indicate significant differences between non-infected and infected mice (student's t-test).

Accumulating evidence suggests that a specialized T cell subset, the T_FH_, is responsible for provision of co-stimulation to B cells in the germinal centre [44], [45]. T_FH_ can be identified by expression of the chemokine receptor CXCR5 and the regulatory receptor programmed death 1 (PD1), in addition to activation markers such as CD44 [46]. DNP-KLH vaccination into the hind footpad did not change the frequency of total CD4^+^ T cells in draining compared to non-draining lymph nodes (Figure 7B). However, the frequency of T_FH_, defined as PD1^+^CXCR5^+^ cells within the CD4^+^CD44^+^ population (Figure 7A), increased selectively in the lymph node draining the site of vaccination (Figure 7C). The strict correlation of T_FH_ expansion within the stable CD4^+^ compartment to the site of vaccination in both, non-infected and nematode-infected mice, strongly suggests that these T_FH_ were generated in response to vaccination.

Draining lymph nodes of mice that were infected with *L. sigmodontis* for 60 days at the moment of vaccination displayed a significant reduction in both, T_FH_ frequency within activated CD4^+^ T cells and absolute T_FH_ numbers in comparison to non-infected vaccinated mice (Figure 7C). Further characterization of T_FH_ induced in non-infected and nematode-infected mice revealed no significant differences in the expression of inducible co-stimulator (ICOS) or Foxp3 (Figure 7DE). Thus reduced numbers of DNP-specific B cells in DNP-KLH-vaccinated nematode-infected mice were correlated with reduced numbers of vaccination-induced T_FH_ whereas the phenotype remained comparable.

### Prolonged interference with vaccination efficacy after termination of *L. sigmodontis* infection

**Figure 8:**
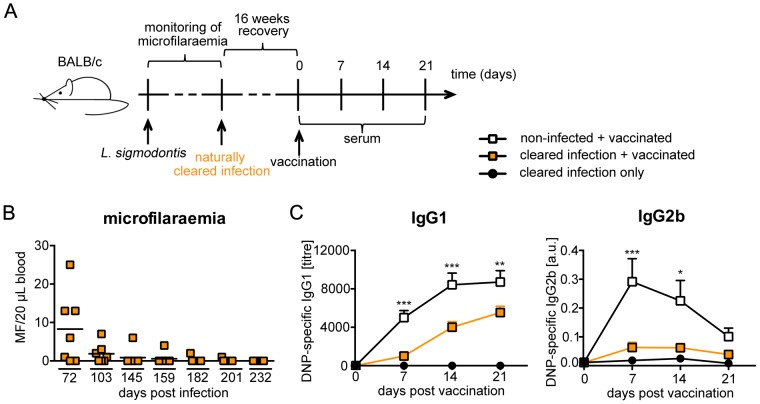
Suppressed IgG response to TD vaccination 16 weeks after termination of *L. sigmodontis* infection. **(A)** Diagram of the experimental setup. Six- to eight-week-old BALB/c mice were naturally infected with *L. sigmodontis* (closed squares and circles) or left non-infected (white squares). Mice were allowed to naturally clear the infection. Microfilaraemia was monitored and the time point no MF was detectable in the blood of any previously infected mouse was defined as termination of infection. 16 weeks later non-infected mice (open squares, n = 14) and mice, which had terminated the infection (orange squares, n = 15) were vaccinated with 100 µg DNP-KLH/Alum i.p. An additional control group was allowed to clear the infection, but was not DNP-KLH/Alum-vaccinated (closed circles, n = 2). **(B)** Microfilaraemia of mice naturally infected with *L. sigmodontis* of one experiment representative for three independent repeats. **(C)** DNP-specific IgG1 and IgG2b was quantified in sera. Results are expressed as mean ± SEM of pooled data derived from three independent experiments. Asterisks indicate significant differences of the mean of DNP-specific Ig in non-infected and infected mice (Two-way ANOVA).

The collective data presented above suggest that vaccinations may fail in individuals carrying chronic nematode infections due to impaired induction of T_FH_. To evaluate the kinetics of this nematode-induced suppression, immune competent BALB/c mice were first allowed to terminate *L. sigmodontis* infection and then vaccinated with DNP-KLH (Figure 8). Immune-mediated control of infection was indicated by clearance of MF from the circulation that was observed between day 180 and day 280 p.i. (Figure 8B and data not shown). Responses to vaccination were still reduced in mice that were previously nematode-infected when vaccination was carried out immediately after termination of microfilaraemia and four or eight weeks after clearance of MF from the circulation (data not shown). Therefore we finally introduced an additional recovery period of 16 weeks after clearance of microfilaraemia before vaccination was performed (Figure 8A). Strikingly both, IgG1 and IgG2b responses to DNP-KLH vaccination were still significantly suppressed in mice that had terminated *L. sigmodontis* infection at least 16 weeks before vaccination (Figure 8C). Thereby DNP-specific IgG2b responses were almost absent in mice with a history of previous *L. sigmodontis* infection.

It should be noted that although clearance of MF from the circulation at these late time points of infection strongly suggests impaired fitness of the adult parasites due to immune-mediated control and extermination, it does not confer direct information about the presence and the status of adult parasites in the thoracic cavity at the moment of vaccination. No living parasites were detectable in 100% of infected and vaccinated mice three weeks after vaccination (data not shown). We observed limited amounts of remaining dead material in the thoracic cavity of some mice and we cannot formally exclude contribution of this helminth-derived material to suppression of vaccination.

## Discussion

In this study we demonstrate that chronic *L. sigmodontis* infection prevents humoral responses to bystander antigen vaccination. DNP-KLH-vaccinated and nematode-infected mice displayed reduced titres of DNP-specific IgG in the serum and reduced numbers of DNP-specific B cells in the lymph nodes compared to vaccinated, non-infected mice.

*L. sigmodontis*-induced suppression was restricted to TD vaccination and not observed during T cell-independent B cell vaccination using the TI-2 antigen NIP-Ficoll. In congruence with our previous study performed in semi-permissive, day 14 *L. sigmodontis*-infected C57BL/6 mice [31], this study strongly suggests that nematodes suppress antibody-producing B cells indirectly via suppression of accessory T cells. Within the CD4^+^ T cell population a specialized T cell subset, the T_FH_, is central for the initiation of classical B cell responses [44], [45]. Next to PD1 expression, T_FH_ are further characterized by the continued expression of the chemokine receptor CXCR5 that regulates their localisation within the B cell follicle [46]. Separating the sites of nematode- and vaccine-induced immune responses we distinguished between nematode- and vaccine-induced T_FH_. *L. sigmodontis*-infected mice displayed significantly reduced numbers and frequencies of vaccine-induced T_FH_ in the lymph nodes draining the site of vaccination. Interestingly, we did not detect differences in the phenotype of T_FH_ regarding the expression of ICOS, a central co-stimulatory receptor for B and T cell interaction that is essential for antibody responses to TD antigens [47]. Follicular regulatory T cells (T_FR_) that arise from thymus-derived Foxp3^+^ T_reg_ and display a T_FH_-like, CXCR5^+^ PD1^+^ phenotype have been implied in regulation of TD B cells responses via limitation of T_FH_ and B cell numbers in the germinal centre [48], [49]. As Foxp3^+^ expression in T_FH_ was unchanged and depletion of Foxp3^+^ T cells did not abrogate nematode-induced suppression, we ruled out a significant contribution of T_FR_ to nematode-induced immune suppression.

We have shown before that transient gastrointestinal nematode infection predominantly suppressed T_H_1-associated IgG2 responses to vaccination [33], whereas *L. sigmodontis* infection of semi-permissive C57BL/6 mice [31] as well as chronic infection of fully susceptible BALB/c mice in the current study induced a generalized suppression of both T_H_1- and T_H_2-associated isotypes. We also did not observe differences in the affinity of vaccine-induced IgG in nematode-infected and non-infected mice. Taking these findings into account, we hypothesize that nematode infection interfered with the humoral response to vaccination already at the stage of T_FH_ induction. Reduced numbers of vaccine-induced T_FH_ will result in reduced provision of co-stimulation for vaccine-specific B cells. As a consequence reduced numbers of vaccine-specific B cells expand in the draining lymph node leading to reduced titres of vaccine-specific IgG in the peripheral circulation of nematode-infected mice. This hypothesis is supported by our previous study where we reported reduced proliferation of ovalbumin-specific TCR transgenic OT-II T cells as a simplified model for accessory T cells upon adoptive transfer into *L. sigmodontis*-infected mice [31].

Regarding the mechanism, we provide evidence that suppression of humoral response did not reflect direct competition between nematode- and vaccine-specific lymphocytes by separating the sites of nematode- and vaccine-specific immune responses. Suppression of OT-II T cell proliferation during acute *L. sigmodontis*-infection of C57BL/6 mice was shown to be T_reg_-independent and partially mediated by IL-10 [31]. Early depletion of CD4^+^CD25^+^ T_reg_ improved host defence in *L. sigmodontis*-infected BALB/c mice, suggesting an implication of T_reg_ in immune evasion for this genetic background [36]. Since we recently described a central role for Foxp3^+^ T_reg_ in gastrointestinal nematode-induced immune evasion in BALB/c mice that was not functional in C57BL/6 mice [39], it was conceivable that Foxp3^+^ T_reg_ would contribute to the observed suppression of TD vaccination in *L. sigmodontis*-infected BALB/c mice. However, in the current study we show that suppression of IgG response during chronic infection of BALB/c mice was clearly established in the absence of Foxp3^+^ T_reg._ This suggests that immune modulation during acute and chronic *L. sigmodontis* infection in C57BL/6 and BALB/c mice is established by similar mechanisms such as IL-10 induction [31]. Potential mediators of suppression in addition to Foxp3^+^ T_reg_ are IL-10 producing Foxp3^−^ T cells [50], IL-10 producing regulatory B cells [51], [52], alternatively activated macrophages [53], and tolerogenic dendritic cells [54] that have been shown to mediate suppression during nematode infection in several murine systems. We are currently testing the function of these regulatory cell populations in suppression of OT-II T cell proliferation during acute *L. sigmodontis*-infection of C57BL/6 mice.

Using fully susceptible BALB/c mice we dissected the impact of different *L. sigmodontis* life stages on vaccination efficacy. Suppression was induced by L4, immature and mature adults, but not by recently transmitted L3. Injection of isolated MF, in contrast, elevated IgG2a responses to vaccination, thus delivering a pro-inflammatory stimulus. A comparable pro-inflammatory stimulation was mediated by isolated MF in a model of LPS-induced sepsis [55]. In line with one previous study [56], we demonstrate that this pro-inflammatory effect of MF was dominated by the anti-inflammatory effect exerted by *L. sigmodontis* adults. By contrast, the MF-mediated aggravation of LPS-induced sepsis was not rescued by presence of adults but also induced by implantation of mature adults releasing MF [55]. The different outcome may reflect the impact of long exposure (i.e. 60 days) of the host to *L. sigmodontis* before MF occurred in the circulation in our study in contrast to sudden implantation of MF releasing adults. Prolonged exposure may be needed for complete immune modulation to silence the strong pro-inflammatory effect of MF. In line with this reasoning we observed that suppression of DNP-specific IgG response to vaccination clearly increased with duration of infection.

As our study focused on the first response to vaccination we used the early humoral and cellular response as indicator of efficacy. In order to model vaccination efficacy for the human situation more precisely, also the magnitude of memory responses several months after initial vaccination in absence and presence of helminth infection will be compared in future studies.

Suppression of vaccination responses, once established, was observed several months after immune-mediated termination of infection. We cannot exclude that remaining helminth-derived material in the thoracic cavity contributed to suppression in the mice that had cleared microfilaria from the circulation 16 weeks before vaccination was performed. However, comparable remnants of large parasites are likely to be present in humans with a history of previous filarial infection as well. Thus, the prolonged suppression of vaccination-induced responses reported in this study may have implications for health policy. While some murine studies suggest that drug-induced termination of helminth infection may improve vaccination efficacy after 1–3 weeks of recovery [21], [22], our results show that the immune status does not immediately return to normal responsiveness in a setting modelling chronic infection. Prolonged suppression of vaccination responses after drug-induced termination of infection have been described in other mouse models. However, in these studies responsiveness was eventually achieved after recovery periods of 8 and 16 weeks, respectively [57], [58]. Regarding the human population in the tropics where nematode infections are endemic a re-infection during such a recovery period is likely to occur. Despite the limits of murine models to reflect every aspect of the human situation, combined evidence gained in different mouse models for helminth infection can be informative. Since first murine studies demonstrated successful vaccination despite concurrent nematode infection by improved vaccination strategies [21], [30], [59], [60], we suggest that development of vaccination regimes that are functional despite pre-existing nematode infection would be more promising and should be considered in addition to deworming programs before vaccination.

## Supporting Information

Text S1 **Figure S1.** *L. sigmodontis* infection reduces quantity but not quality of DNP-specific IgG. Six- to eight-week-old BALB/c mice were naturally infected with *L. sigmodontis* (blue squares, n = 10) or left non-infected (white squares, n = 10) and vaccinated with 100 µg DNP-KLH/Alum i.p. at day 60 p.i. Control mice (black circles, n = 2) were infected but not vaccinated. DNP-specific IgG1, IgG2a and IgG2b was quantified in sera of mice. Results are expressed as mean ± SEM of pooled data derived from two independent experiments. Asterisks indicate significant differences of the mean of DNP_38_-specific Ig in non-infected and infected mice (Two-way ANOVA). **Figure S2.** *L. sigmodontis* infection suppresses humoral response to TD vaccination in the presence and absence of MF. Quantification of DNP-specific IgG1, IgG2a and IgG2b in sera of DNP-KLH-vaccinated non-infected (white squares, n = 12) and *L. sigmodontis*-infected **(A)** microfilaraemic mice (blue squares, n = 7) and **(B)** non-microfilaraemic mice (blue squares, n = 12). Results are expressed as mean ± SEM of pooled data derived from four independent experiments. Asterisks indicate significant differences of the mean of DNP-specific Ig in non-infected and infected mice (Two-way ANOVA). **Figure S3.** *L. sigmodontis*- specific Ig response during infection. Six- to eight-week-old BALB/c mice were naturally infected with *L. sigmodontis* and not vaccinated (black circles). Non-infected mice (open squares) and infected mice (grey squares) were vaccinated with 100 µg DNP-KLH/Alum i.p. *L. sigmodontis*-specific Ig in the sera was quantified by ELISA at the indicated time points post infection. Results are expressed as mean ± SEM of pooled data derived from at least five independent experiments (days 14 and 102: n = 4, days 30, 60 and 81: n = 6).
(PDF)
