# Supplementary material for: Nematode-Induced Interference with Vaccination Efficacy Targets Follicular T Helper Cell Induction and Is Preserved after Termination of Infection
Source: PLoS Negl Trop Dis. 2014 Sep 25;8(9):e3170. doi: 10.1371/journal.pntd.0003170 (PMC4177885; doi:10.1371/journal.pntd.0003170)
Supplement: Text S1 — Figure S1. L. sigmodontis infection reduces quantity but not quality of DNP-specific IgG. Six- to eight-week-old BALB/c mice were naturally infected with L. sigmodontis (blue squares, n = 10) or left non-infected (white squares, n = 10) and vaccinated with 100 µg DNP-KLH/Alum i.p. at day 60 p.i. Control mice (black circles, n = 2) were infected but not vaccinated. DNP-specific IgG1, IgG2a and IgG2b was quantified in sera of mice. Results are expressed as mean ± SEM of pooled data derived from two independent experiments. Asterisks indicate significant differences of the mean of DNP38-specific Ig in non-infected and infected mice (Two-way ANOVA). Figure S2. L. sigmodontis infection suppresses humoral response to TD vaccination in the presence and absence of MF. Quantification of DNP-specific IgG1, IgG2a and IgG2b in sera of DNP-KLH-vaccinated non-infected (white squares, n = 12) and L. sigmodontis-infected (A) microfilaraemic mice (blue squares, n = 7) and (B) non-microfilaraemic mice (blue squares, n = 12). Results are expressed as mean ± SEM of pooled data derived from four independent experiments. Asterisks indicate significant differences of the mean of DNP-specific Ig in non-infected and infected mice (Two-way ANOVA). Figure S3. L. sigmodontis- specific Ig response during infection. Six- to eight-week-old BALB/c mice were naturally infected with L. sigmodontis and not vaccinated (black circles). Non-infected mice (open squares) and infected mice (grey squares) were vaccinated with 100 µg DNP-KLH/Alum i.p. L. sigmodontis-specific Ig in the sera was quantified by ELISA at the indicated time points post infection. Results are expressed as mean ± SEM of pooled data derived from at least five independent experiments (days 14 and 102: n = 4, days 30, 60 and 81: n = 6). (PDF) [file pntd.0003170.s001.pdf]

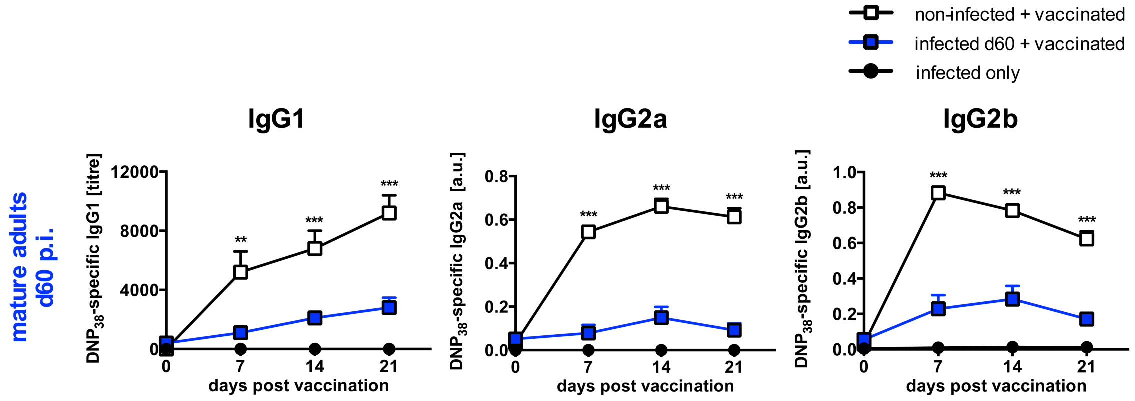

Supplementary Figure S1: *L. sigmodontis* infection reduces quantity but not quality of DNP-specific IgG.

Six- to eight-week-old BALB/c mice were naturally infected with *L. sigmodontis* (blue squares, n=10) or left non-infected (white squares, n=10) and vaccinated with 100 µg DNP-KLH/Alum i.p. at day 60 p.i. Control mice (black circles, n=2) were infected but not vaccinated. DNP-specific IgG1, IgG2a and IgG2b was quantified in sera of mice. Results are expressed as mean ± SEM of pooled data derived from two independent experiments. Asterisks indicate significant differences of the mean of DNP_{38}-specific Ig in non-infected and infected mice (Two-way ANOVA).

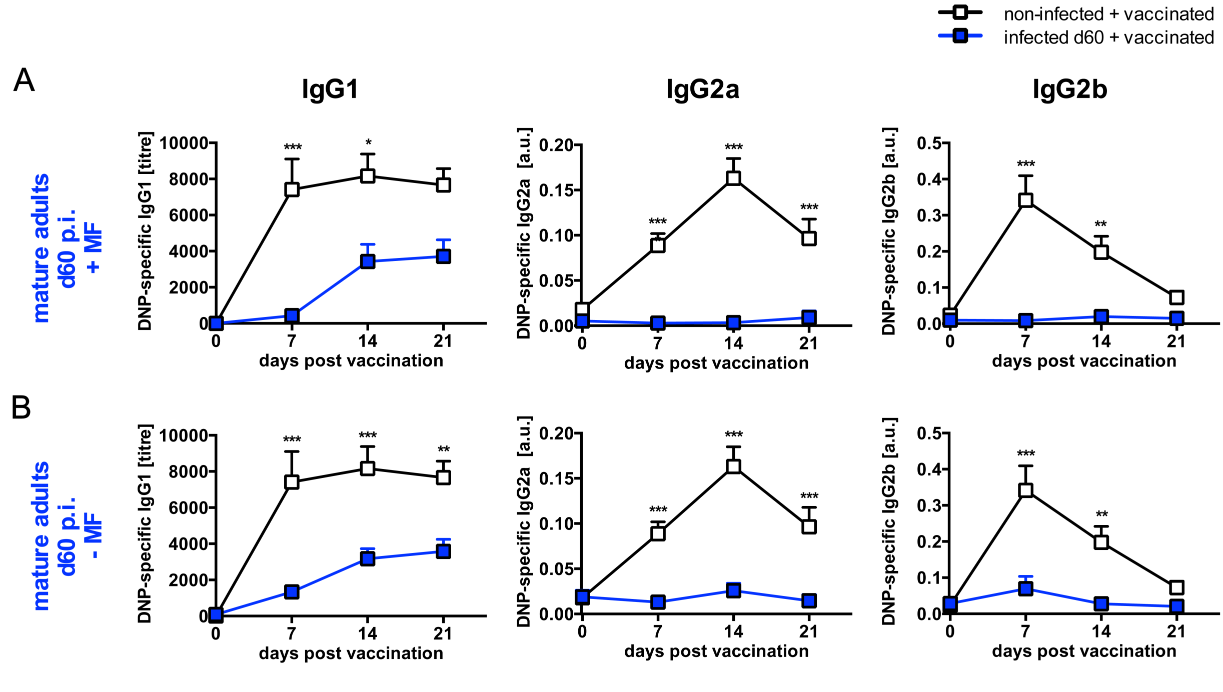

Supplementary Figure S2: *L. sigmodontis* infection suppresses humoral response to TD vaccination in presence and absence of MF.

Quantification of DNP-specific IgG1, IgG2a and IgG2b in sera of DNP-KLH-vaccinated non-infected (white squares, n=12) and *L. sigmodontis*-infected **(A)** microfilaraemic mice (blue squares, n=7) and **(B)** non-microfilaraemic mice (blue squares, n=12). Results are expressed as mean ± SEM of pooled data derived from four independent experiments. Asterisks indicate significant differences of the mean of DNP-specific Ig in non-infected and infected mice (Two-way ANOVA).

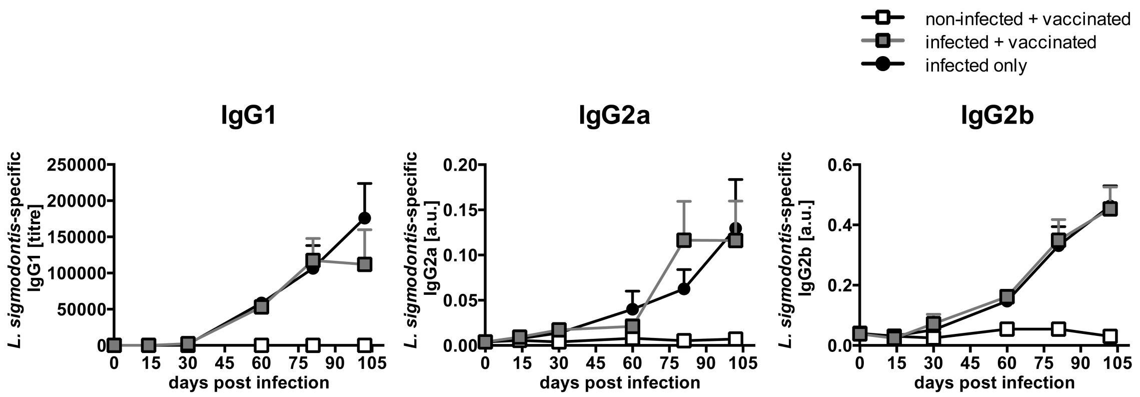

Supplementary Figure S3: ***L. sigmodontis*-specific Ig response during infection.**

Six- to eight-week-old BALB/c mice were naturally infected with *L. sigmodontis* and not vaccinated (black circles). Non-infected mice (open squares) and infected mice (grey squares) were vaccinated with 100 µg DNP-KLH/Alum i.p. *L. sigmodontis*-specific Ig in the sera was quantified by ELISA at the indicated time points post infection. Results are expressed as mean ± SEM of pooled data derived from at least five independent experiments (days 14 and 102: n=4, days 30, 60 and 81: n=6).
